# Progress in Low-Impact Processing Technologies to Deliver More Sustainable and Healthy Food Tomorrow

**DOI:** 10.3390/foods14132332

**Published:** 2025-06-30

**Authors:** Marco Dalla Rosa, Santina Romani, Pietro Rocculi, Urszula Tylewicz, Silvia Tappi

**Affiliations:** 1 Department of Agriculture and Food Sciences, Alma Mater Studiorum University of Bologna, Piazza Goidanich 60, 47521 Cesena, Italy; santina.romani2@unibo.it (S.R.); pietro.rocculi3@unibo.it (P.R.); urszula.tylewicz@unibo.it (U.T.); silvia.tappi2@unibo.it (S.T.); 2 Interdepartmental Centre for Agrifood Industrial Research, Alma Mater Studiorum University of Bologna, Via Quinto Bucci 336, 47521 Cesena, Italy

**Keywords:** non-thermal technologies, sustainability, healthy food, ultra-processed food

## Abstract

Following the debate on food processing, resulting in a negative definition of ultra-processed products, the improvement of the food system could be pursued through the co-creation of new food solutions aimed at enhancing human health and increasing safety and sustainability, in particular by using neglected foodstuff, crops or by-products, and applying mild processing technologies. The proper management of mild/non-thermal processing technologies, such as dynamic and hydrostatic high-pressure, vacuum impregnation, ultrasound, pulsed electric field and cold plasma applications, can result in a less negative effect with respect to the traditional thermal treatments, and, in some cases, the overall functionality can be improved. In many cases, these treatments can induce structural changes that improve the bioaccessibility and/or the bioavailability of bioactive compounds such as probiotic microorganisms. Moreover, non-thermal pretreatments, also combined with mild thermal drying technology, could lead to a significant reduction in the total request of energy, even when considering the energy input for their application. A selected review of results published in the last few years on those strategies is presented, considering studies carried out within the frame of different national and EU projects.

## 1. Introduction

### 1.1. Challenges

As is well known, the agri-food production system is called to tackle different challenges. UN recommendations have also been developed following Food and Agriculture Organization of the United Nations (FAO) alerts on food production sustainability, which have been published in some FAO reports, in particular that published in 2011 on Livestock in Food security [1]. Since the publication of this report, there has been concern about the environmental sustainability of actual food habits in so-called developed countries, considering that 75% of available lands are already used for animal production, and 18% of global gas emissions (14.5% of which result from human activities) are related to animal (cattle and ruminants in general) production.

Moreover, these concerns might even increase, as the global population is projected to reach 9.6 billion by 2050, about 1.4 billion more than today, together with increasing urbanization, thus making it more and more difficult to access locally produced food, mainly in developing countries [2]. Furthermore, the increase in the world’s population will be accompanied by an increase in the middle class in those countries where economic development is higher; purchasing power goes hand-in-hand with an increasing demand for richer food, both from a quality and nutritional point of view.

Therefore, it is necessary to provide evidence of the need for change. Firstly, to guarantee the availability of sufficient and secure food for all in the future; to accomplish this, scientists, geneticists, agronomists and engineers shall be involved in actions to tackle climate change and resource reduction in order to pursue a “sustainable intensification” of food production. Furthermore, the relationship between food/nutrition and sustainability in a rational approach is related to food losses and wastes. Nowadays, it is well known that around 30% of global food production is lost or wasted along the food chain, from production to the consumer’s dish [3]. Farmers, growers, food producers and processors are mainly involved in introducing technological innovations to reduce food losses from the field, where raw materials are produced, along with the food chain and distribution [4].

### 1.2. Nutrition Needs

Recently, the European Food Safety Authority (EFSA) published an update of the Dietary Reference Values for the EU, which includes recommended values for microelements like sodium and chloride, which are examples of the challenges posed by the Reference Intake Levels of Nutrients and Energy (LARN) recommendations when there is a complex physiological interaction between several nutrients. The updated LARNs have been differently designed; other than different recommendations for the general population, it includes those for infants (7–11 months), children and adolescents (1–17 years), adults (≥18 years), pregnant women and lactating women [5]. Following these recommendations, the Healthy Eating Pyramid was created, addressing other aspects of a healthy lifestyle (weight control and regular exercise, vitamin D and multivitamin supplements, moderate alcohol consumption, etc.), and is therefore a useful tool for health professionals and health educators. Another tool is the Healthy Eating Plate, which can be used to create a balanced meal by following a few simple recommendations. The meal should consist of vegetables and fruits (^1^/_2_ of your plate), whole grains, which should be preferred (^1^/_4_ of your plate), protein sources (^1^/_4_ of your plate) and healthy plant oils, such as olive oil, in moderation [6].

### 1.3. Food Production and Environment

Owing to the aforementioned advice, the recommendation to drastically reduce meat consumption emphasizes the need to explore new sources of protein that are suitable for healthily supplying the human body with the required proteins and nutrients. Furthermore, a swift global food transformation towards healthy diets from sustainable food systems is even more necessary, and without such a food transformation, the world will not meet the targets set in the United Nations SDGs and the Paris Climate Agreement. In fact, the agri-food system is among the major drivers of climate change and other critical climate factors, such as changes in land use, the depletion of freshwater resources and the pollution of both terrestrial and aquatic ecosystems; Ref. [7] analyzed several options in order to reduce the environmental effects of the food system, including dietary changes towards healthier diets, with high quantities of plant-based products, improvements in technologies and management, and reductions in food loss and waste. Their analysis showed that it may be possible to stay within planetary boundaries, considering the environmental pressure expected by 2050, with a combination of high-ambition measures for greenhouse gas emissions and nitrogen and phosphorus applications, and with a combination of medium-ambition measures for cropland and blue water use. Analyzing the options at the planetary level shows in detail the possible combinations of the different measures. The analysis showed that an ambitious dietary change toward a more plant-based and flexitarian diet is needed to stay within the midpoint of the greenhouse gas (GHG) limit, in combination with other factors like food loss and waste reduction, and technological improvements, especially to stay within the mean values of cropland and blue water boundaries [7,8]. On the other hand, there is a need for technological improvement due to the necessity to achieve a reduction in energy and production factors so to increase food processing sustainability. Some innovative processing technologies, based on non-thermal physical principles, promise a lower energy input in food processing, thus combining the “mild” treatment of food with a better utilization of energy and water resources, which should lead to more sustainable processes in terms of the environmental impact. The proper validation of the range of these emerging and novel technologies is, however, needed through accurate and complete robust data collection so to ensure the full reliability of the introduction of these new technologies.

### 1.4. Food Classification

Consumers demand foods that are healthy and palatable, but which are also able to satisfy the needs linked to changing lifestyles in an increasingly complex relationship between work, interests, different activities and the time to prepare food.

In this context, the food industry is increasingly moving towards offering processed foods to provide safe, healthy and palatable food with longer service and shelf-life characteristics to be consumed outside of the home or at home with reduced preparation time. Nevertheless, in recent years numerous epidemiological studies have highlighted the relationship between industrial products, generically considered as “processed” or “ultra-processed” (UPF), and health risks. The increase in obesity and other food-related problems (e.g., cardiovascular disease and diabetes) has been related to the intensity of processing according to the NOVA classification [9,10]. A great debate has started around the topic, with different viewpoints held between nutritionists, food scientists and food engineers; however, it appears quite clear to the majority of the food science community that only classifying foods according to the level of processing, considering the number of ingredients, is not only wrong but also misleading, and could be an obstacle for the food industry to move forward with innovations [11]. To this end, all functional foods will be classified as UPFs [12]. In fact, from one side, consumers view UPFs negatively, while new food trends demand fewer ingredients, “clean labels” and “clean eating” diets [13]. However, the food processing industry could use the occasion of the “processing debate” to change the narrative in those consumers that view these products negatively, even while new food trends demand fewer ingredients, “clean labels” and “clean eating” diets. Many food companies can already point to ongoing initiatives around product reformulation. A greater attention to food processing could spur innovation in food processing technology, as well as the introduction of novel, low-impact technologies, even if adding one or more processing steps [14].

A conceptualization of processed foods was drawn by [15], where processed foods were divided as a function of the following different aspects: the extent and nature of change, as well as the place and purpose of processing. From the analysis of the literature on UPFs and NOVA classifications, and the assumption of the effect on consumers’ health, and considering the viewpoint of food science and technology, processing and nutritional value do not have a linear relationship, and these concepts need to be dissociated [15]. Furthermore, the impact of UPFs on greenhouse gas emissions seems to be similar to that produced with minimal processing. The advancements in food processing technologies can affect this impact on the entire supply chain, thus reducing the potential threat to sustainability and biodiversity [12].

## 2. Role of Innovation to Introduce Low-Impact Technologies

Following the increasing demand for healthy and functional food, in combination with enhancing food system sustainability, intensive research efforts have been made to develop new processing technologies as an alternative to conventional thermal processing in order to obtain safe products, with respect to conventionally processed products, with sensory and nutritional properties much more like those of fresh products [16]. The validation and potential implementation of non-thermal technologies in the food production chain, including agri-food waste, to enhance the processing efficiency at lower treatment intensities were reviewed by [17].

In a basic semantic structure, we could imagine a relationship between minimal processing throughout mild and non-thermal (bio)technologies —> the co-creation of new food solutions —> the enhancement gut health and the increase in safety and sustainability.

### 2.1. Cold Atmospheric Plasma

Cold atmospheric plasma (CAP) technology is based on the ionization of a gas mixture that leads to the formation of excited molecules, ions, electrons and radical species that co-exist with electromagnetic radiation (UV and visible light). It is considered an innovative technology that can be applied in food processing for decontamination and stabilization in the food and packaging industry [18,19]. Recently, research on the application of cold plasma has been focused much more on different aspects than decontamination, including toxicant degradation, enzymatic inactivation, functionalization, quality improvement and nutrient extraction [20,21].

In the frame of a collaborative research project (Italian National Project PRIN PLASMAFOOD), a system composed by a high-voltage generator, a treatment chamber where you can set the temperature and the operating gas, was realized. Finally, there is the plasma source itself, as Surface Dielectric Barrier Discharge (SDBD), with optical absorption spectroscopy to monitor the process, in order to find a connection between the efficacy of our treatment and the concentration of different reactive species. The equipment was developed in collaboration with Alma Plasma, a spin-off of the University of Bologna (https://site.unibo.it/idea/en/our-innovative-businesses-start-ups-and-spin-offs/almaplasma-srl (accessed on 26 June 2025)) [22].

Cold plasma treatment was used first as a decontamination treatment to extend the food shelf-life, like in the case of fresh-cut fruit [16,23], where the microbial growth and enzymatic activities of fresh-cut melon, apples and kiwifruit were studied. The results of yeasts, lactobacilli and lactococci on melon samples demonstrated that the tested cold plasma treatment was very promising in order to stabilize fresh-cut melon samples, allowing for efficient decontamination. Furthermore, a significant increase in microbial shelf-life was observed by optimizing the treatment time, which was due to delayed growth during the storage of the surviving spoilage microflora. Another very interesting approach to assess the cold plasma effect on product quality was the use of isothermal calorimetry (TAM) in order to evaluate the metabolic response to stress in fresh-cut tissue through the determination of metabolic heat production. The results regarding this approach showed that the heat production of the treated samples was significantly lower compared to the controls for all 24 h of the analysis, proportionally to the treatment time. The calculation of the total metabolic heat produced by the fruit tissues during storage at 10 °C and the differences among the samples were more pronounced after 24 h of analysis compared to after 12 h. Considering the enzymatic activity, a slight reduction was observed, but this effect was dependent on the type of enzyme considered, since peroxidase (POD) and pectin methylesterase (PME) activities were assessed. Cold plasma was also used for virus decontamination, both as gas and as plasma-activated water (PAW). Hepatitis A virus (HAV) and Noroviruses, using murine Norovirus (MNV) as a surrogate for human Noroviruses, were tested. The viral stocks used were HAV strain HM175 and MNV strain MNV-1. Reduced infectivity and viral integrity PCR (EMA-rt-q-PCR) were evaluated [24] according to the literature [25]. As a consequence of the experience carried out on foodborne viruses, during the COVID-19 pandemic, the application of CAP was forwarded on the decontamination of SARS-CoV-2 from food and food packaging surfaces. CAP treatment for 10 min completely degraded the inoculated SARS-CoV-2 RNA molecules on packaging materials and packaged products, whose concentration resulted below the detection limits for each target sequence as detected by the RT-PCR reaction performed using the n-COVID Allplex SARS-CoV-2 assay mix [22].

The application of CAP to reduce enzymatic activity was also studied; Ref. [26] carried out research on the inactivation of polyphenoloxidase (PPO) in sugar model systems to further elucidate the results already shown in the literature [27,28]. PPO inactivation data after different exposure times to CAP as a function of the reactive species (O_3_ concentration) owing to the CAP treatments, fitted by a first-order model using a Peleg model, showed that the CAP treatments significantly (*p* < 0.05) reduced the PPO activity, with inactivation depending on the processing time (*p* < 0.05) and ozone concentration in the treatment chamber. Spectroscopic analyses revealed that the loss of the PPO activity was due to a change in the protein tertiary structure and the loss of alpha-helices structures [26].

Cold plasma has been applied to decontaminate fish and seafood products, generally considered as the most perishable foodstuffs, but which is also particularly sensitive to quality change during plasma treatments [29,30,31,32,33]. In general, the application of cold plasma caused a reduction from 1 to 3 log CFU/g of naturally present or inoculated microorganisms on fishery products. The effect depended on the process parameters and the type of the microorganism to be inactivated. However, the negative effect of cold plasma on these products, rich in highly unsaturated fatty acids, is lipid oxidation, which could increase in the treated products due to the highly oxidative power of plasma reactive species [31]. In the framework of the FutureEUAqua EU project, Ref. [34] investigated the effect of cold plasma generated (SDBD system) with different gas mixtures on the safety, quality and nutritional aspects of fresh sea bream fillets. The gas mixtures used were air (80% N_2_ and 20% O_2_) and Argon (80% Argon and 20% O_2_), and *E. coli* and *L. innocua* were investigated as inoculated microorganisms. The most effective plasma treatment appeared to be Argon-20, which had the highest mortality rate for all microorganisms, with a reduction slightly higher than 1 log CFU/g, with *E. coli* being the most sensitive microorganism, although the differences were not statistically significant with the treatment with Air-20. Fatty acids (FAs) and protein quality were tested in both not-digested and digested samples (according to the INFOGEST^®^ protocol) to assess the bioaccessibility of FAs. Regardless of the treatment, no differences in the fatty acid composition were detected between the control and treated samples, and the only difference emerging from the NMR spectral data was an overall 10% reduction in protein hydrolysis in Air-20 fillets compared to Argon-20 and controls. With the exposure of seafood to CAP, TBARS concentrations significantly increased up to about 3.5–4.0 mg MDA/kg, without significant differences among the samples, and this result agrees with most of the literature. Since lipid oxidation might affect the sensorial properties of the product, a useful strategy to protect CAP-treated seafood from lipid oxidation, other than the optimization of processing conditions, could be the application of natural antioxidant compounds before treatment.

A further enforcement of the use of cold plasma has been the research on the reduction in mycotoxins in intermediate moisture and dried food so to enhance their healthiness; Ref. [35] investigated the efficacy of cold atmospheric plasma (CAP) treatment in degrading the emerging *Alternaria* toxins as pure molecules and as natural contaminants in dried tomatoes. The effects of CAP treatments under O_3_ and NO_x_ regimes on naturally contaminated dried tomatoes with low and high tenuazonic acid contaminations (TeA, the most abundant *Alternaria* mycotoxin found in naturally contaminated products) were the reduction in toxins by about 40% after 15 min and up to 55% after 60 min of treatment. The 15 min treatment on dried tomatoes artificially contaminated at two concentration levels of TaA led to a more than 20% and more than 35% toxin reduction according to the initial toxin concentration, confirming that a higher substrate concentration resulted in a greater mycotoxin degradation. Lower effects have been found on other *Alternaria* toxins, such as alternariol (AOH), alternariol monomethyl ether (AME), altenuene (ALT) and tentoxin (TEN). Thus, the results showed that CAP treatment can be an effective method to reduce different *Alternaria* mycotoxins in sun-dried tomatoes, ensuring food safety and reducing mycotoxin contamination in agricultural products.

As previously described, recently, cold plasma treatments were studied to obtain further objectives than decontamination. Following this approach, Refs. [36,37] studied the modification of the functional properties of starches of different origins affected by plasma-activated water (PAW) treatments in order to enhance their technological suitability. Using PAW treatments of potato starch in combination with annealing (ANN) showed a synergistic effect in terms of the thermal stability and shear resistance, leading to a lower breakdown viscosity, higher elasticity and gelatinization enthalpy, probably due to crosslinking. Modified potato starch obtained using those treatments could be used as a more sustainable material in food applications for baked goods, as well as a thickening agent for candies and ice cream, and which is generally used in many processed foods, as they are better able to withstand the high thermal and mechanical processing conditions in the food industry. In addition, the PAW and ANN treatments can significantly enhance the behavior of potato starch in terms of water interaction and the resulting functional properties. Finally, studies on the functional properties of the pasting properties of normal maize, waxy maize and potato starches subjected to PAW treatments throughout the deep rheological analysis of three starches, characterized by different botanical origins and different compositional and structural properties, led to very interesting results. Cereal and tuber/roots starches can be successfully modified by PAW, resulting in a promising strategy for starch modification as a “green” alternative for existing methods of starch modification in the food industry. The results obtained in those studies, however, show that the degree of modification is strongly dependent on the starch type (the botanical, amylose and amylopectin content). Therefore, process optimization based on the specific substrate appears necessary for obtaining tailored functionality.

### 2.2. Pulsed Electric Field (PEF)

The exposure of biological cells to an external electric field affects the membrane permeability; the application of short electric pulses (pulsed electric field (PEF) treatment) causes a phenomenon called electroporation, when the formation of transient or permanent pores occurs, leading to an impairment of the membrane semipermeability [38,39,40]. Depending on the level of the applied electric field with an intensity above a threshold value, the electroporation can be reversible (transient pores) or irreversible (permanent pores), so that permanent pores are formed and the membrane is damaged, leading to cell death [41,42]. The electric field strength is typically in the range from 0.5 to 1.5 kV/cm for reversible electroporation, and from 1.0 to 5.0 kV/cm for irreversible permeabilization, in plant or animal tissues. The irreversible electroporation (with an electric field strength of 20–50 kV/cm) is also widely applied for enzyme and microorganism inactivation [43]. Nevertheless, in the last years more work has been performed on the benefit that PEF technology as reversible electroporation can give to mass and heat transfer, and on the recovery on bioactive compounds from by-products or neglected products, both crops and animal products, including seafoods.

An example of the use of low-intensity PEF to improve the mass and heat transfer is reported by [16], as research led in the context of the FP7 ERA-Net CORE Organic Plus project.

Reversible PEF was applied in combination with a mild process like the osmotic dehydration (OD, where water removal occurs without any state change at ambient temperature) of organic strawberry, considering mass process transfers and water distribution, as assessed by means of low-frequency Time-Domain Nuclear Magnetic Resonance (TD-NMR). Regarding the mass transfer improvement, the results showed an increase in water and solid kinetics due to the permeabilization of the cell membranes induced by the PEF treatment. The application of an electric field intensity of 100 V cm^−1^ was already sufficient to increase the water loss by 12% after 60 min of osmotic dehydration. PEF treatment effects should be considered time-dependent; the formation and growth of pores in the membrane are not immediate but continue for several minutes after the treatment. With regard to the water distribution owing to osmotic dehydration, TD-NMR showed a decrease in the mean transverse relaxation time (T2) values of the water populations during the osmotic treatment due to the water removal and the different water–solute–biopolymer interactions. In the PEF-treated samples, a fast T2 decrease was observed immediately after the treatment due to the different water–solute–biopolymer interactions induced by the loss of compartmentalization within the strawberry tissue. Thus, the results showed that the OD efficiency could be highly improved by the PEF pretreatments by facilitating the diffusion of the inner water by affecting the membrane permeability. Finally, the highest quality properties, as determined by the firmness and color, were obtained using a trehalose and CaCl_2_ solution as the osmotic agent. Cell viability was partially maintained in the strawberry samples treated with an electric field strength intensity of 100 V cm^−1^.

In [17], the combination of PEF and osmotic dehydration treatments prior to the final air-drying process (led at low–medium temperatures) to obtain shelf-stable products using kiwifruit waste (undersized fruits) has been reported. Different mathematical models were used to fit the drying kinetics, and the results showed that the PEF/OD-treated sample had higher drying kinetic rates, presenting better quality and acceptability as measured from both instrumental and sensorial analyses.

The combination of PEF with mild air drying was performed on vegetables by [44], applying PEF pretreatments prior to low–medium-temperature air drying (40°, 55° and 70 °C). The results showed an acceleration of the drying kinetics from 32% to 48%, according to the drying temperature, to reach the final target water activity (a_w_ = 0.30) in the dried samples of black cabbage. In carrots, the application of PEF before convective drying at 70 °C reduced the drying time by 6.9–8.2% in comparison with the untreated material [45]. In addition, better color retention was recorded in the PEF-treated samples in comparison with the untreated dried samples.

As mentioned above, the application of low-intensity PEF to achieve reversible electroporation has been widely used to recover bioactive compounds from by-products or neglected products. Following this strategy, the application of PEF was studied to improve bioactive compound recovery from brewery by-products [17,46]. Response surface plots were used to show the combined effects of process variables for free phenolic compound and flavan-3-ol extraction from brewers’ spent grains (BSGs). The obtained optimal PEF conditions (2.5 kV/cm, 50 Hz and 14.5 s) improved the yield of free and bound phenolics by 2.7 and 1.7 times, respectively, compared to the control samples without PEF treatment. The total phenolic content (free and bound) obtained in brewers’ spent grains after the PEF treatment at optimal conditions (640.46 μg g^−1^ d.w.) was 43.23% higher than in the control sample (363.58 μg g^−1^ d.w.). These phenolic extracts could be useful as ingredients in the food industry because of the low cost and high nutritional value of BSGs [47]. These extracts could be used to enrich bakery products such as bread, biscuits, cookies and pasta products.

Another interesting application of PEF to recover functional compounds from neglected sources is to improve the extraction of chitin, chitosan and carotenoid from seafood by-products [48]. In fact, chitosan has been studied extensively in the last few years due to its interesting properties as an antioxidant, emulsifying agent, ingredient in edible and active films/coatings, flocculating and clarifying agent, and food preservative, having antimicrobial and antifungal properties. In food formulation, chitosan can be used as food fiber, in the immobilization of enzymes and as a stabilizer of color, texture and odor [49,50]. PEF has been used to improve the recovery of chitin and chitosan, which is its deacetylated form, from crustacean by-products [51], following the methodological approach to apply non-thermal technology—including ultrasound, high-pressure processing, pulsed electric fields, cold plasma and supercritical fluid technology—in the extraction of valuable components from wastes and by-products. In fact, crustacean by-products obtained from the processing plant, namely, heads, shells, pleopods and tails, contain different valuable compounds, such as chitin and chitosan, carotenoids, lipids and proteins.

Bioactive compounds or products, obtained from crustacean by-products to be used in seafood products, have been listed by [49]. Extracted compounds, mainly chitosan, from the shells of shrimp, crab and prawn, as well as the shrimp cephalothorax, head and tail, are used in fish sticks, while croaker fish and surimi have different functions, from antioxidants to cryoprotectants and gelling agents. Carotenoid astaxanthin recovery from Red (*Aristeus antennatus*) and Camarote (*Melicertus kerathurus*) shrimp side streams were studied using PEF in combination with accelerated solvent extraction (ASE) in order to valorize a highly valuable by-product but with a general reduction in processing waste [50]. The PEF treatment led to a significant increase in the extraction yield when followed by solvent extraction. The recovery of astaxanthin in *M. kerathurus* and *A. antennatus* increased by 46% and 48%, respectively, compared to the control. Better results were obtained using dimethyl sulfoxide (100% DMSO) with respect to absolute ethanol as a solvent. Similarly, the Trolox equivalent antioxidant capacity (TEAC) and oxygen radical absorbance capacity (ORAC) of shrimp side stream extracts also showed higher activities with the PEF–ASE combination. Nevertheless, these technologies are not yet adapted to the reality of the shellfish processing industry, and extensive research is still needed to find the technological and economic conditions for their implementation. The advantages and disadvantages of PEF technology in extraction processes are reported in Table 1 [49].

Reversible PEF can promote metabolic stress responses, and therefore influence the production of secondary metabolites in plant products and cell cultures. Generally, higher polyphenol, carotenoid and anthocyanin contents during the storage of pumpkin, apples, berries, tomato and other vegetables as stimulation due to the reversible PEF treatment, as well as an increase in glucosinolates in *Brassicaceae*, was found, probably also due to the increase in the extractability [53,54,55].

Stimulating the biosynthesis of plant secondary metabolites with antioxidant and other biological properties associated with important health benefits through PEF could be an interesting opportunity for the food industry to meet the increasing demand for foods with a high functional value.

In the case of olives, corn seeds and soybeans, an increase in the yield of oil in olives, and an increased recovery of isoflavonoids in soybean oil and phytosterols in maize germ oil were found, as reviewed by [56].

The application of PEF on seed germination has been reviewed by [57]; reversible PEF treatments were applied on the seeds of barley, wheat, wheatgrass and others, with a very wide range of operating conditions. Among the specific effect on different species, considering wheat seeds, monopolar rectangular pulses with different frequencies, treatment times and total energy were found to be an alternative to chemical treatments for surface disinfection, significantly increasing the germination and seedling rates by 10 and 28%, respectively, compared to the untreated sample, and the PEF treatments allowed for tolerance to cold and salt stress, together with an improved vigor. All of the reviewed literature hypothesizes that, as the main mechanism, PEF can improve seed germination and the growth rate by stimulating a physiological response throughout ROS production and oxidative stress. Nevertheless, the proper mechanism is still far from being fully understood.

**Figure 1:**
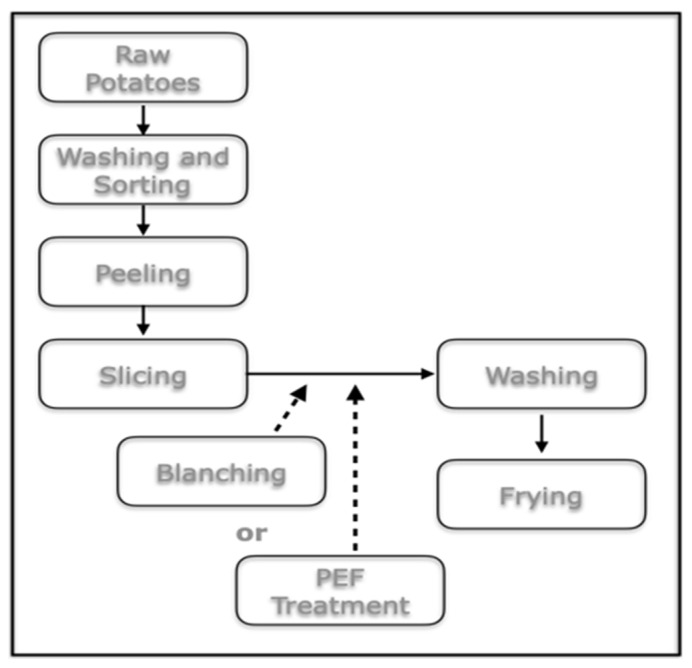
PEF treatment in the pre-frying of potato stick processing (from [58]).

A further successful application of PEF was tested by [58,59], with the application of PEF treatment to reduce the formation of acrylamide (AA) in potato during frying (Figure 1).

Electroporation was better able to reduce the reagents in the raw material, as sliced potato, in comparison with the blanching treatment (48% vs. 40% reduction, respectively), having a higher effect on the reduction in free asparagine than reducing sugars. On the other hand, blanching—as a thermal treatment—would change other quality properties, such as the texture behavior, during frying, so that would possibly be avoided. Acrylamide reductions were found to be around 30% and 17% in the PEF and blanched potato slices after frying. In another study, Ref. [59] performed a combination of PEF and yeast pre-treatments for sliced potato prior to frying, as it was shown that the yeast *Aureobasidium pullulans* L1 strain can successfully reduce AA formation due to the enzymatic and metabolic activity of the yeast, which is able to reduce the levels of glucose, fructose and asparagine [60]. Nevertheless, the yeast pretreatment alone led just to a slight AA reduction (around 5%), while the yeast treatment combined with PEF, followed by water dipping, was the most effective in reducing AA, leading to a reduction of around 36% and 59%, respectively, for a 5 and 15 min dipping time. Thus, the combination of these non-thermal (bio)technologies significantly increased the safety of a popular processed food like potato crisps.

### 2.3. Ultrasound Treatment (US)

Ultrasound (US) is a form of energy generated by sound waves with a frequency above the hearing threshold of the human ear (18–100 MHz). In particular, high-intensity (power) ultrasound operates at the frequency range of kHz (18–100 kHz) and waves of high intensity (>1 W/cm^2^) [61,62]. The principles of the application of US are based on the fast compression and expansion of the treated material (the sponge effect) and cavitation phenomena, which consist of the formation of large bubbles moving from growth to collapse, causing the liberation of a high quantity of energy. Both phenomena, the compression/expansion of the tissue and cavitation, promote the disruption of cell walls and membranes, resulting in the formation of microscopic channels in the biological material, thereby promoting the alteration of the physical and chemical properties of food products [63]. Thus, ultrasound-assisted extraction (UAE) is commonly used nowadays in food technology as a substitute for conventional extraction techniques so to improve the recovery of bioactive compounds from plant materials, but also to increase food safety, since US can be successfully used in food enzymes and microbial inactivation [16,64]. A list of US applications in fruit and vegetable products subjected to different processing parameters is reported in Table 2. These data are a synthesis of selected results reported in the cited publications highlighting the US treatments of various fruits and vegetables in terms of improving the bioactive compound content, and thus the potential impact on consumer health, which have been widely reviewed in the literature [65,66,67]. In particular, flavonoids undoubtedly have great potential to prevent/treat many chronic diseases associated with oxidative stress and inflammatory components.

It is well established that ultrasound treatment can increase the mass transfer during the osmotic dehydration of different plant tissues, but the complete overview of the effects that this technology can have on the quality of a specific fruit, like kiwifruit, was largely missed. The study of the selected chemical and physical properties of differently treated kiwifruit is described by [81]. The water activity, freezable water content, texture, color and chlorophyll content of ultrasound-treated kiwifruit were investigated. In addition, the effect of combined treatments (US pretreatment and OD process) on the above-mentioned physico-chemical kiwifruit characteristics was also studied. Generally, the obtained results allow us to claim that US reduced the dehydration time and reduced the oxidation of bioactive components exceptionally at lower temperatures due to the thermolabile character of most substances. Some nutrients can be lost in the liquid phase during the preliminary US treatment. Nevertheless, in most cases, the influence of US pretreatment has been shown in dried products, where the untreated samples exhibited lower quality attributes than the US-treated samples [81]. Ultrasound-assisted osmotic dehydration was also applied to cranberries, where a 30 min sonication at 21 kHz and a total power generated by sonotrodes of 180 W, corresponding to an intensity of 3.6 W/g, was used before the OD treatment was carried out, using sucrose and trehalose as an osmotic solution [81]. The results showed that the best mass transfer values in terms of water removal were obtained in cranberry samples subjected to the combination of OD with sucrose and US treatment. Furthermore, US pretreatment led to a lower weight reduction in samples during storage if treated with any osmotic solution in comparison to those without US pretreatment. The US pretreatment also promoted benefits when considering qualitative characteristics, for example, in the color lightness maintenance, compared to the untreated fruits. Going more deeply into the mechanism of the influence of US treatment on the water state and quality properties, US- and OD-processed cranberry fruits (whole and cut) were studied by differential scanning calorimetry (DSC), SEM microscopy and TD-NMR measurements. While whole fruits demonstrated only a slight effect of the ultrasound treatment, owing to the very tough skin of cranberry, the OD process of cut fruits showed a larger action of the US treatment on the mass transfer. US-assisted OD samples were shown to release water from the vacuoles into extracellular spaces through TD-NMR analysis, and the uptake of sugar as mass transfer from the solution to the fruits showed a significant increase by sonication.

As previously cited, ultrasound-assisted extraction (UAE) has also largely been studied to increase the yields of functional compounds extracted from by-products, underestimated raw material or neglected products [82]. In [74], as an example, the optimal conditions for the application of UAE to the extraction of phenolic compounds from *Psidium guajava* L. leaves were studied. Using Response Surface Methodology (RSM), it was possible to design optimized extraction processing conditions by combining the ethanol/water ratio and US power as processing factors, with flavonols, flavan-3-ols, DPPH, TEAC and the sum of phenolic compounds (SPCs) as dependent factors. This is very interesting to highlight, as the optimal conditions found by RSM could be different, even significantly different, when taking into account the different functional compounds or properties, as reported in Table 3. These results are very promising because the increase in flavonoids has the great potential to prevent/treat many chronic diseases associated with oxidative stress and inflammatory components, such as cardiovascular diseases, cancer, mental health, gastrointestinal diseases and others [66].

### 2.4. Dynamic/Hydrostatic High-Pressure Processing

High-pressure (HP) advanced technologies are promising processing technologies that could by widely used in the food industry due to their valuable features.

High hydrostatic pressure (HHP) consists of the treatment of food products, which are packed and placed in a liquid-filled pressure chamber at a high pressure of 100–1000 MPa at chilled or mild process temperatures (<45 °C), in order to achieve microbial inactivation, modification and extraction. During the treatment, the high pressure compresses the food, causing a reduction in the volume, which counteracts the external forces through the food’s chemical structure [83,84]. Conversely, high-pressure homogenization (HPH) is a food processing technique that uses high pressure to reduce particle sizes and inactivate microorganisms, thus improving product stability and safety. HPH involves forcing a liquid food product through a small valve at high pressures (typically 100–300 MPa). The intense shear forces, turbulence and cavitation are then created in the liquid food. These forces break down large particles and droplets into smaller, more uniform sizes, resulting in a more stable emulsion or suspension [85,86,87]. 

Recently, significant research efforts have been devoted to studying the development and the optimization of HP homogenization and HP hydrostatic processes [88,89]. Nevertheless, the impact of the high-pressure effect on the nutritional properties for poorly investigated raw material is still unknown, even though it is generally considered that, when compared to a corresponding thermal treatment, HHP seems to reduce vitamin loss and improve its maintenance during storage. In orange juice pressed at 100–400 MPa from 30 to 60 °C up to a 5 min treatment time, an increase in the hesperidin concentration as a function of the applied pressure was observed, but not for flavanones. After 10 min of treatment of HHP at 400 MPa on apple juice, an increase in hydroxycinnamic, procyanidin acid and catechin contents was reported, as reviewed by [16]. 

A metabolomic approach to evaluate HPP/HHP treatments has been performed by [90] on minced gray mullet (*Mugil cephalus*) during chilled storage. Samples pasteurized by high hydrostatic pressure (HHP) at 0, 400, 500 and 600 MPa were analyzed using proton NMR-based metabolomic techniques, alongside microbial analysis. HHP showed bacteriostatic properties reducing the bacterial counts and a positive influence on the formation of volatile compounds, thus demonstrating the efficiency of HHP as a non-thermal technology to preserve fish freshness. These findings highlight HHP as an efficient approach for preserving the freshness of fish, which is also related with the elimination of common seafood pathogens, such as Vibrio and Listeria spp., and slowing the growth of spoilage microorganisms [90]. Concerning the effects on the bio-metabolisms, HHP was able to significantly reduce (*p* < 0.05) the production of spoilage-related molecules such as TMA, tyramine, hypoxanthine and ethanol. In fact, the key metabolic pathways, a part of the lysine metabolism, were influenced by the HHP treatment. The results showed the ability of the NMR-based metabolomics strategy to effectively identify processing effects on food matrices. Very interestingly, there was a schematic presentation of the effect of the HHP treatment on the different metabolism pathways, such as the amino acid, carbohydrate, pyruvate, nitrogen and nucleotide metabolisms, and on the level of efficacy in stimulating the increase in metabolite concentrations extracted from different treatments. Nevertheless, further research and development are needed to find a preservation method able to inhibit the lysine metabolism, as pointed out by [91].

**Figure 2:**
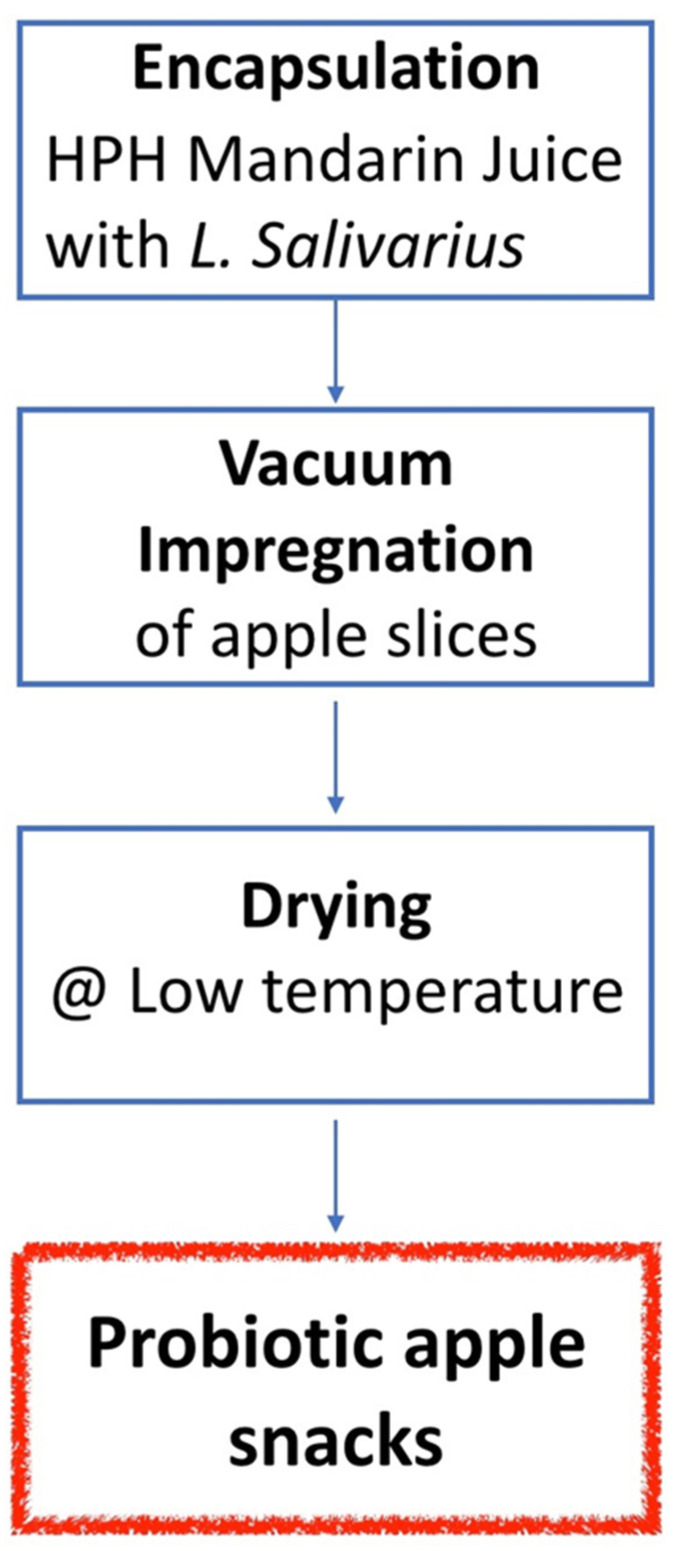
Flow diagram of processing to obtain a snack with impregnation of citrus juice with HPH-encapsulated probiotic *Lactobacillus salivarius* (from [93]).

High-pressure technologies are also used in dynamic processes for fluid food in the form of high-pressure homogenization (HPH). The application of this technology can impact both the stability, with partial sanitation prolonging the product shelf-life, and the improvement of the nutritional, sensorial and textural properties of foods, like juices, beverages, emulsions and purees. On the other hand, although phenolic compounds have a key role in the health benefits of fruit juice consumption, little is known about the effect of processing on their bioaccessibility; Ref. [92] reported on the impact of high-pressure homogenization processing at 20 MPa on the nutritional and functional value of mandarin juice with and without the addition of trehalose and probiotic *Lactobacillus salivarius*. During digestion, there is a release of phenolic compounds from the food matrix, which is an important prerequisite for their effectiveness in the human body, and so it is essential to find technological treatments that not only maintain the concentration of phytochemicals, but also their bioaccessibility. HPH treatments were shown to reduce the concentration of total phenolics and the main flavonoids, but also to significantly increase their bioaccessibility after in vitro digestion, probably due to the disruption of cells and membranes, which can increase the availability of functional compounds. Further positive results were related to the total antioxidative capacity (TAC) not reduced by the HPH treatment, and the effect of the treated juices against ROS generation and lipid oxidation in cultured liver cells (HepG2 cells) in a basal condition. HPH-treated citrus juices with the addition of a probiotic were the most protective samples both in terms of the accessibility of bioactive molecules and the concentration of reactive oxygen species (ROS) among stressed cells [40]. Research regarding the addition of probiotics was also carried out to study the effect of the HPH treatment as an encapsulation process that is able to give further functionality to citrus juice with microencapsulated probiotic microorganisms to be used in the vacuum impregnation of the fruit matrix; Ref. [93] investigated the probiotic survival and in vitro digestion of encapsulated *Lactobacillus salivarius* spp. *salivarius* included into an apple sample by vacuum impregnation, dried by low-temperature air drying and stored for 30 days (Figure 2).

The encapsulation of the probiotic was performed by HPH at 70 MPa on an emulsion to obtain microcapsules, and added to mandarin juice, with vacuum impregnation at 50 mbar for 10 min. Drying was carried out at 40 °C to reach a water activity value of around a_w_ = 0.50. The experimental design resulted from the conceptualization of the strategy of research previously defined as improving the food functionality by studying the structure–property relationships [93]. The survival of inoculated microorganisms throughout the process to obtain the dried impregnated apple samples was assessed on *L. salivarius* with and without microencapsulation, resulting in a significantly better survival in the samples with the microencapsulated probiotic; however, in both cases, an evident reduction (from around 8 Log CFU/g to around 7 Log CFU/g of dried fruit) was due to the drying process, even though it was carried out at a low temperature. During storage, after 30 days, the microencapsulation was able also to protect the probiotic from degradation phenomena in the dried apple samples (with a percentage of survival of 39% in comparison to 19% for the non-encapsulated probiotic). The simulated gastrointestinal digestion of dried apple samples encapsulated and non-encapsulated with *L. salivarius* showed that the encapsulated probiotic had a higher resistance, and the number of *L. salivarius* spp. *salivarius* in the impregnated and dried apple was enough, thus maintaining that the specific functionality of *L. salivarius* is mainly a potential effect against *Helicobacter pylori* infection.

Furthermore, Ref. [94] demonstrated that the application of 200 MPa (ultra-high-pressure homogenization, UHPH) for three cycles made it possible to obtain a stable kiwifruit juice in refrigerated storage for more than 40 days, and increased the shelf-life by 1 week at room temperature compared to the control, while at the same time increasing the availability of polyphenols and the antioxidant activity, and better preserving the color. In fact, HPH promoted the disarrangement of the cell clusters into single cells and/or cell fragments. Storage at three temperatures from 5 to 25 °C also showed microbial load under limits at non-refrigeration temperatures (15 and 25 °C), with a shelf-life of 9 and 5 days, respectively. From a rheological viewpoint, the release and solubilization of cell wall constituents, such as pectin and proteins, caused the increase in the volume fraction of particles and led to the improvement of particle interactions, thus increasing the viscosity. The obtained results on the antioxidant capacity (TEAC) agree with the previous literature, where the UHPH process can improve the extractability of antioxidant components through the disruption of the cell wall components. A UHPH treatment of 200 MPa for three cycles showed a slight decrease in the TPC in kiwifruit juice. On the other hand, an increase in the initial antioxidant activity was observed in the samples treated by HPH, and this might be explained by a partial inactivation of polyphenoloxidase and peroxidase enzymes, thereby causing the degradation of phenolic compounds in the vegetable matrix.

## 3. Conclusions

Non-thermal pretreatments, such as cold plasma, pulsed electric fields, high-pressure processing and ultrasound processing, also combined with mild thermal drying technology, would bring a significant reduction in the total request of energy, even when considering the energy inputs for their application, as reported by several authors in the literature and as reviewed by [95]; the advantages in terms of the quality and carbon footprint reduction are summarized in Table 4.

Dynamic and hydrostatic high-pressure, vacuum impregnation, ultrasound, pulsed electric field and cold plasma applications can result in a less negative effect with respect to traditional thermal treatments, and the overall functionality can also be improved by recovering functional components from by-products or wastes, following the circular bioeconomy approach.

**Figure 3:**
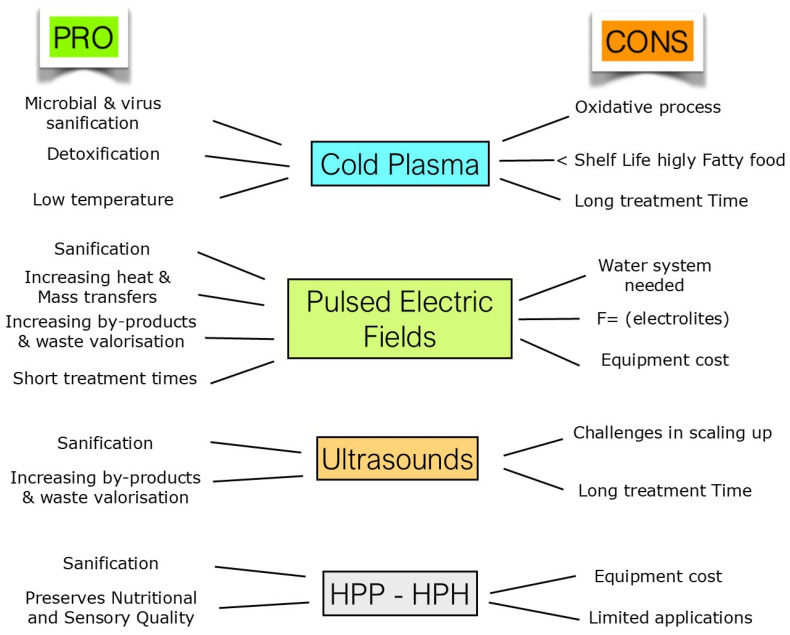
Comparison of pros and cons of investigated technologies.

In Figure 3, a comparison of the positive and negative properties of the technologies considered is reported.

These treatments can produce structural changes that improve the bioaccessibility and/or the bioavailability of bioactive compounds, such as probiotic microorganisms, to improve food healthiness and gut microbioma.

Non-thermal low-impact processing technologies could help the food industry to pursue a better recognition in sustainability, and to offer better food functionality, thereby recovering favorable consideration in the framework of the ultra-processing debate.

However, an interdisciplinary approach among food engineers, microbiologists, food chemists, bio-NMR specialists and nutritionists is necessary to research the best processing conditions, and to increase and assess the sustainability of processing in the food industry.

## Figures and Tables

**Table 1:** Advantages and disadvantages of PEF technology in comparison with traditional extraction technologies [49,52].

Technology	Advantages	Disadvantages
	Non-thermal behavior	Poorly studied for the compound’s extraction from crustacean by-products
	High selectivity	Possible limited utilization due to the conductivity of matrix
PEF	Less time and energy consumption	High initial investment of PEF equipment
	High yields for carotenoid extraction	Limited extraction of lipophilic compounds
	Does not require any additional chemicals	
	Can be used in continuous mode	

**Table 2:** Ultrasound (US) application in fruit and vegetable products subjected to different processing parameters (modified from [16]). TPC = total phenolic content.

Product	US Parameters	Results Obtained by US Application	References
Acerola juice	18 kHz, 2000 to 3000 W/L	Increase in the availability of pro-vitamin A and vitamins B3, B5, C and E	[68]
Carrots	20 °C, 120 min	Increase in the extraction rate of TPC (2186 mg of gallic acid/100 g_dw_), vitamin C (148 mg/100 g_dw_) and β-carotene (12 mg/100 g)	[69]
Carrots	21 and 35 kHz frequency for 10, 20 and 30 min	Increase in the carotenoid content at 35 kHz	[70]
Cashew apple bagasse	226 W/cm^2^, bagasse/water ratio of 1:4 (*w*/*w*), 6 min	Increase in the extraction of vitamin C	[71]
Cranberry	21 kHz, 180 W, 30–60 min	Retention of Vit. C with 30 min, while loss with longer treatment Good retention of polyphenols, anthocyanins and flavonoids for both durations	[72]
Cranberry	21 kHz, 180 W+ blanching	Increase in bioactive compounds such as Vit. C, polyphenols, anthocyanins and flavonoids	[72]
Green tea	22–83 °C, tea-to-water ratio (12–73 g L^−1^), amplitude (23–77%)	Max. polyphenol content (12,318 mg L^−1^) and max. flavonoids (3774 mg L^−1^) at 77 °C, tea-to-water ratio of 73 g L^−1^ and amplitude of 77%	[73]
Guava leaves	24 kHz, 200 W, 40 min, ethanol/water (*v*/*v*) ratio of 60%	Increase in the extraction of flavonols and flavan-3ols	[74]
Lemon by-products	150–250 W, 45–55 °C, 35–45 min	Optimal TPC extraction of 18.10 ± 0.24 mg GAE/g_dw_ at US power of 250 W, 50 °C and 45 min Optimal rutin extraction of 3.20 ± 0.12 mg/g_dw_ at US power of 150 W, 48 °C and 35 min	[75]
Peaches	37 kHz, 10–30 min, 30–50 °C, ultrasonic power of 30–70%	The optimal conditions for extractions of TPC were 41.53 °C, 43.99% and 27.86 min	[76]
Pumpkins	37 kHz, 10–30 min, 30–50 °C, ultrasonic power of 30–70%	The optimal conditions for extractions of TPC were 41.45 °C, 44.60% and 25.67 min	[76]
Sour cherry	25 kHz (0.4 W/cm^2^), 30–120 min	At short times, no effect on bioactive compounds; prolonged application provoked loss of about 10% of total polyphenols	[77]
Spinach	37 and 80 kHz, 5–30 min, 30–50 °C, ultrasonic power of 30–70%	Max. total phenol (33.96 ± 11.30 mg gallic acid/g_dw_) and flavonoids (27.37 ± 11.85 mg/g_dw_) at 37 kHz, 30 min, 40 °C and 50%	[78]
Strawberry	40–70 °C and 30 and 60 W	>65% of Vit. C retention in dried product	[79]
Sweet potato	28 kHz, 300 W, 20–60 min	>70% of Vit. C retention in osmodehydrated product; however, higher loss of carotenoids	[80]

**Table 3:** Optimal conditions obtained by RSM for ultrasound-assisted extraction of different functional compounds/properties from guava leaves (adapted from [74]).

RSM Optimal Conditions	DPPH (μmol Trolox/g Leaf d.w.)	TEAC (μmol Trolox/g Leaf d.w.)	SPC (mg/g Leaf d.w.)	Flavonols (mg/g Leaf d.w.)	Flavan-3-ols (mg/g Leaf d.w.)
Time (min)	22	45	41	38	37
EtOH/water ratio (% (*v*/*v*))	54	58	62	62	63
US power (W)	80	180	230	235	228

**Table 4:** Effects on food quality and carbon footprint elements of non-thermal processing (adapted from [95]).

Effects on Food Quality	Carbon Footprint Reduction
Minimal quality loss	Less wastewater
Increasing bioavailability	Increasing energy and water savings
Reduction in processing contaminants	Lower environmental impact
Maintenance of nutritional values	Decreased operational costs
Maintenance of sensorial properties	Decreased electricity
Inactivation of microorganisms	Less time-consuming
Improvement of heat and mass transfer	Inexpensive
Improvement of firmness and texture	Non-hazardous
Decreased color change	Minimal source demands
Increased shelf-life	Simple processing design

## Data Availability

No new data were created or analyzed in this study. Data sharing is not applicable to this article.
